# Pathogenic spectrum, antibiotic resistance, and risk factors for 28-day mortality in pediatric bloodstream infection-associated sepsis: a 10-year retrospective cohort study

**DOI:** 10.1128/spectrum.00194-26

**Published:** 2026-07-20

**Authors:** Qian Han, Xishu Deng, Ruohong Jin, Rong Hu, Min Su, Weibi Li, Tianyi Ma, Honglin Liu, Yanjun Wang

**Affiliations:** 1 Pediatric Intensive Care Unit, Kunming Children’s Hospital, Children’s Hospital Affiliated to Kunming Medical University, Kunming, Yunnan Province, China; 2 Department of Laboratory, Kunming Children’s Hospital, Children’s Hospital Affiliated to Kunming Medical University, Kunming, Yunnan Province, China; 3 Pediatric Outpatient Department of Maternal and Child Health Care Hospital of Dali Bai Autonomous Prefecture, Dali, Yunnan Province, China; 4 Department of Emergency, Kunming Children’s Hospital, Children’s Hospital Affiliated to Kunming Medical University, Kunming, Yunnan Province, China; bioMerieux Inc, Eugene, Oregon, USA

**Keywords:** pediatric sepsis, bloodstream infection, pathogenic spectrum, antibiotic resistance, mortality risk factors

## Abstract

Sepsis induced by a bloodstream infection (BSI) is a common cause of mortality in pediatric intensive care units. Currently, there is a lack of data for sepsis from southwest China. This study involved a 10-year retrospective analysis to summarize pathogen and drug resistance features of sepsis induced by BSI and analyze risk factors for 28-day mortality. A total of 311 pediatric patients with sepsis induced by a BSI were identified with the following characteristics: males, 60.8%; average age, 26 (5.6, 108.0) months; and 28-day mortality rate, 20.3%. The incidence of septic shock and acute kidney injury was 51.8% and 26.0%, respectively. Polymicrobial growth was demonstrated in 11 cultures. A total of 305 pathogens were isolated, as follows: 164 strains (53.8%) were gram-positive bacteria, 125 strains (41.0%) were gram-negative bacteria, and 16 strains (5.2%) were fungi. The most frequently isolated pathogens were *Escherichia coli*, coagulase-negative Staphylococcus, and *Staphylococcus aureus*. Risk factors for mortality were the Phoenix Sepsis Score (PSS) (>5.5), neutropenia, acute kidney injury, and mechanical ventilation. Risk factors for community-acquired sepsis were consistent within the entire cohort. The PSS was a risk factor for hospital-acquired sepsis. *E. coli* had the highest levels of cephalosporin and fluoroquinolone resistance, while *Klebsiella pneumoniae* had the highest level of carbapenem resistance. In conclusion, it is crucial to monitor multidrug resistance in the local geographic area and select antibiotics based on the results of drug sensitivity tests.

**IMPORTANCE:**

This study provides crucial 10-year data for pediatric sepsis from bloodstream infections in southwest China, a previously underrepresented region. The local pathogen spectrum was identified, revealing high rates of multidrug resistance in key gram-negative bacteria, like *Escherichia coli* and *Klebsiella pneumoniae*. Furthermore, critical risk factors for 28-day mortality, including the Phoenix Sepsis Score, neutropenia, acute kidney injury, and mechanical ventilation, were noted. These findings are vital for clinicians and facilitate early risk stratification, guide empirical antibiotic therapy based on local resistance patterns, and ultimately enhance management strategies to reduce mortality in this vulnerable population. The data fills a significant regional knowledge gap and has direct implications for improving pediatric sepsis outcomes.

## INTRODUCTION

Pediatric sepsis is a life-threatening condition triggered by severe inflammatory responses to infection and is a major cause of morbidity and mortality in pediatric intensive care units (PICUs). The prevalence of pediatric sepsis is up to 8% among PICU patients, and the mortality rate ranges from 7.8% to 40% (1). The mortality rate in children with severe sepsis is as high as 34.6% in China (2). Several factors are related to the prognosis of children with sepsis, including a reduced platelet count, an elevated lactate level, a bloodstream infection (BSI), multiple organ dysfunction syndrome, acute kidney injury (AKI), and septic shock (3, 4). Among these factors, the incidence of BSIs in pediatric sepsis has been increasing (10%–30%) (5). Multiple studies have identified BSI as a significant concern among critically ill patients. Indeed, a BSI in an ICU patient can significantly prolong the hospital stay and increase medical costs (6–8). Furthermore, the widespread use of antibiotics has led to a gradual increase in the incidence of antibiotic-resistant bacteria. Inappropriate use of antibacterial drugs can lead to an increase in the incidence of multidrug resistance (MDR) and the mortality rate (9). Therefore, measures should be implemented as a priority to prevent such infections in the neonatal ICU and pediatric PICU.

However, the distribution of pathogenic bacteria in a BSI among patients from different geographic regions varies greatly, which makes early intervention for a BSI challenging (6). The most common bacteria in a BSI include *Escherichia coli*, *Staphylococcus aureus*, coagulase-negative Staphylococcus (CoNS), and *Klebsiella pneumonia*e. The prevalence of these bacteria depends on patient demographics and geographic region (10). Notably, the prevalence of BSIs varies according to infection source, specifically community-acquired sepsis (CAS) versus hospital-acquired sepsis (HAS) (5, 11). Understanding the clinical features and distribution of pathogens in BSI-induced sepsis among children is essential for treating severely infected children.

In this study, the risk factors for 28-day mortality were identified, and the pathogen spectrum and drug resistance associated with BSI-induced sepsis were characterized. Moreover, the disparity between CAS and HAS is described.

## MATERIALS AND METHODS

### Study design and patient inclusion

A 10-year retrospective cohort study was conducted that included children with sepsis caused by a BSI who were admitted to the PICU of Kunming Children’s Hospital (Kunming, China) between June 2015 and June 2025. The diagnosis of sepsis was based on the 2024 Phoenix Sepsis Score (PSS) International Consensus (12). The inclusion criteria were as follows: (i) 29 days to 14 years of age; (ii) PSS ≥2 points; and (iii) positive blood culture. The exclusion criteria were as follows: (i) isolated organisms identified as contaminants; (ii) incomplete clinical data >20%; and (iii) positive result from the culture of the catheter tip. The study design flowchart is shown in Figure S1.

### Microbiologic methods and definitions of resistance phenotypes

Blood samples were collected from the peripheral veins or central venous catheters of the children. Each sample (volume, 1–5 mL) was immediately inoculated into an aerobic/anoxic culture bottle, then transported to the laboratory. Blood cultures were processed using the BD BACTEC FX 400 fully automatic bacterial culture system (Becton, Dickinson and Company, Franklin Lakes, NJ, USA). Drug sensitivity tests were performed using the VITEK2-CompAct fully automatic bacterial identification and drug sensitivity analyzer (bioMérieux, Marcy-l'Étoile, France) for routine minimum inhibitory concentration tests. Susceptibility testing was performed using standard methods. The breakpoints for all antibiotics, except tigecycline, followed the guidelines in the annual editions (2015–2025) of the Clinical and Laboratory Standards Institute M100. Breakpoints were not retrospectively revised for isolates tested in earlier years. The European Committee on Antimicrobial Susceptibility Testing clinical breakpoints were applied to tigecycline. Isolates categorized as intermediate (I) were not categorized as resistant in the analysis.

The following resistance phenotypes were defined: (i) MDR, non-susceptibility to at least one agent in three or more antimicrobial categories; (ii) extended-spectrum beta-lactamase-producing phenotype, confirmed by the double-disk synergy test (ceftazidime/clavulanate and cefotaxime/clavulanate); and (iii) methicillin-resistant *S. aureus* (MRSA), oxacillin MIC ≥4 µg/mL or cefoxitin disk diffusion zone ≤21 mm.

The incidence rates of four major drug resistance phenotypes were investigated, as follows: difficult-to-treat resistance (DTR); fluoroquinolone resistance (FQR); carbapenem resistance (CR); and extended-spectrum cephalosporin resistance (ECR). The definitions were as follows: DTR, resistant or intermediate resistance to all β-lactam drugs, including carbapenems and fluoroquinolones; FQR, resistance to ciprofloxacin or levofloxacin; CR, resistance to imipenem or meropenem; and ECR, resistance to ceftriaxone or cefepime but not including natural drug resistance.

### Data collection

Demographic and clinical data were collected for children with sepsis within 24 h of diagnosis and included the following: (i) baseline characteristics, such as age, gender, clinical symptoms, and underlying diseases; (ii) laboratory indicators, such as hematological index, C-reactive protein (CRP) level, and procalcitonin (PCT) level; (iii) pediatric critical illness score (PCIS), the arterial partial pressure of oxygen-to-fraction of inspired oxygen (P:F) ratio, the pediatric sequential organ failure assessment (pSOFA) score, and the PSS; (iv) microbiologic results, including blood culture results and antibiotic-resistance phenotypes; and (v) occurrence of complications and intervention measures, including continuous renal replacement therapy and mechanical ventilation (MV). The 28-day in-hospital mortality rate and length of hospital stay (LOS) were recorded. Sepsis is classified into CAS and HAS based on whether it is a nosocomial infection (13).

### Definitions and outcome measurements

Sepsis was defined as a suspected or confirmed infection in a pediatric patient with a PSS ≥2 points. Septic shock was defined as confirmed sepsis in a pediatric patient with a cardiovascular system score of ≥1 point. The definition of pediatric sepsis and organ dysfunction was established according to the recommendations of the 2024 International Pediatric Sepsis Conference (12). The PSS was calculated using the worst values within 24 h of meeting sepsis criteria. The PSS was calculated using the following variables: respiratory (PaO₂:FiO₂ ratio, SpO₂:FiO₂ ratio, and oxygen support); cardiovascular (mean arterial pressure, lactate level, and vasoactive medication use); coagulation (platelet count, international normalized ratio, D-dimer level, and fibrinogen level); and neurologic (Glasgow Coma Scale). All variables were extracted from routine clinical documentation. Although the study period spanned from 2015 to 2025, the PSS criteria were applied uniformly to all patients to ensure harmonization of historical data. The investigators reviewed raw physiologic and laboratory data from medical records to retrospectively calculate the PSS for patients prior to 2024. Patients were included in the sepsis cohort if sufficient clinical and laboratory data were available to retrospectively calculate the PSS. A very small number of patients (<2%) with missing data for multiple PSS components were excluded because the PSS could not be reliably calculated.

An isolate was considered a true pathogen if the following criteria were met: (i) clinical signs and symptoms of sepsis (fever [>38.5°C] or hypothermia [<36°C], tachycardia, tachypnea, or hypotension); and (ii) a typical pathogen (e.g., *S. aureus*, *E. coli*, *K. pneumoniae*, *Pseudomonas aeruginosa*, and *Candida* spp.) from a single blood culture or potential skin contaminants (CoNS) and at least two separate blood culture sets drawn within 48 h were positive. A single positive culture for CoNS without any clinical correlation was classified as a contaminant and excluded from the pathogenic spectrum and resistance analysis.

The main outcome of this study was the 28-day mortality rate of pediatric patients with sepsis induced by a BSI. The secondary outcomes included the LOS and the distribution of pathogens.

### Statistical analysis

Raw data were recorded in a secure electronic database in the form of a variable table. SPSS (version 31.0) was used to perform statistical analyses. Quantitative data that did or did not follow a normal distribution are denoted by mean ± standard deviation and median (IQR) values, respectively. Categorical variables are presented as frequencies (%). Quantitative data of the two groups were analyzed using Student’s t-test or Mann-Whitney U test, and categorical variables were analyzed using a chi-square test or Fisher’s exact probability test.

Candidate variables for multivariable analysis were selected based on a combination of clinical relevance (identified from the literature and clinical judgment) and statistical significance in univariable analysis (*P* < 0.05). Multicollinearity was assessed using the variance inflation factor. The number of events per variable was calculated as the number of 28-day deaths divided by the number of predictor variables in the final model. Multivariable logistic regression analyses were performed to identify independent risk factors. First, the backward LR stepwise method with inclusion criteria (*P* < 0.05) was used for variable selection, and the final independent risk factors were obtained. The selected variables were entered into the final multivariate model using the forced entry method to avoid the overfitting bias of the stepwise method, and internal validation was performed using 1,000 bootstrap resamples to evaluate model stability. The Hosmer-Lemeshow test was used to assess goodness of fit for the model, and a *P* value >0.05 indicated a good fit. The area under the receiver operating characteristic curve (AUC-ROC) was calculated to assess the discriminative performance of the final model. Differences were considered statistically significant when *P* was <0.05 for both sides.

## RESULTS

### Clinical features of pediatric patients with sepsis induced by a BSI

A total of 311 pediatric patients with sepsis induced by a BSI were identified during the study period, as shown in Table 1. The characteristics of the patients were as follows: males, 60.8%; average age, 26 (5.6, 108) months; and 28-day in-hospital mortality rate, 20.3%. There were 157 patients (50.5%) with underlying diseases; hematologic disorders accounted for 85 cases (27.3%). There were 224 children (72%) with respiratory failure, 161 (51.8%) had septic shock, and 81 (26%) had AKI.

**TABLE 1:** Clinical characteristics and outcomes of pediatric BSI bacterial sepsis^*a*^

Items	All patients (*n* = 311)	Group 1			Group 2		
		Survival (*n* = 248)	Non-survival (*n* = 63)	*P* value	CAS (*n* = 200)	HAS (*n* = 111)	*P* value
Gender (male), *n* (%)	189 (60.8)	154 (62.1)	35 (55.6)	0.342	121 (60.5)	68 (61.3)	0.895
Age (mo), *n* (%)	26 (5.6, 108.0)	26 (5.0, 107.7)	25 (7.0, 117.0)	0.441	14 (3.1, 76.3)	84 (17.0, 137.0)	<0.001^*b*^
LOS (days)	24 (11, 38)	27 (16, 43)	4 (2, 24)	<0.001^*b*^	17 (7, 30)	32 (25, 48)	<0.001^*b*^
Underlying condition, *n* (%)	157 (50.5)	124 (50.0)	33 (52.4)	0.736	74 (37.0)	83 (74.8)	<0.001^*b*^
Nosocomial infection, *n* (%)	111 (35.7)	91 (36.7)	20 (31.7)	0.464	/	/	/
PCIS	82 (76, 88)	84 (78, 90)	74 (68, 78)	<0.001^*b*^	80 (74, 86)	84 (78, 90)	<0.001^*b*^
P/F values	200.5 (136.7, 297.7)	219 (150.4, 334.7)	133.3 (90.0, 205.0)	<0.001^*b*^	188 (133.9, 255.4)	247 (143.8, 378.0)	<0.001^*b*^
pSOFA score	7 (5.0, 10.0)	6 (4.0, 8.5)	13 (11.0, 15.0)	<0.001^*b*^	7 (4.0, 10.0)	6 (5.0, 10.0)	0.931
Phoenix score	3 (2, 5)	3 (2, 4)	8 (6, 9)	<0.001^*b*^	4 (2, 6)	4 (2, 6)	0.777
Laboratory test							
WBC (4–10 × 10⁹/L)	8.7 (1.5, 15.9)	9.4 (1.7, 17.6)	4.9 (0.5, 12.1)	0.003^*b*^	11.5 (5.4, 18.3)	1.6 (0.3, 11.9)	<0.001^*b*^
NC (1.0–6.0 × 10⁹/L)	5.1 (0.4, 11.8)	6.5 (0.6, 12.4)	2.6 (0.1, 7.2)	0.004^*b*^	6.9 (2.5, 12.8)	0.5 (0.1, 8.2)	<0.001^*b*^
LC (2.0–9.0 × 10⁹/L)	1.6 (0.5, 3.6)	1.7 (0.6, 3.7)	1.2 (0.3, 3.2)	0.093	2.3 (0.9, 4.6)	0.6 (0.2, 1.6)	<0.001^*b*^
Hb (110–140 g/L)	95.0 (81.0, 109.0)	96.0 (82.0, 109.0)	90.0 (74.0, 111.0)	0.132	96.0 (83.3, 108.8)	88.0 (78.0, 110.0)	0.103
PLT (100–300 × 10^9^/L)	120 (31.0, 306.0)	165.0 (41.0, 343.5)	42.0 (17.0, 130.0)	<0.001^*b*^	168.0 (47.0, 356.0)	46.0 (19.0, 233.0)	<0.001^*b*^
CRP (0–10mg/L)	108.5 (33.1,193.7)	103.2 (29.1, 193.2)	150.3 (54.1, 198.3)	0.091	116.6 (49.2, 200.0)	87.8 (20.4, 171.7)	0.008^*b*^
PCT (0–0.25ng/mL)	8.0 (1.3, 46.5)	6.3 (0.9, 41.6)	19.3 (4.5, 71.5)	0.004^*b*^	9.5 (1.8, 64.4)	4.9 (0.8, 22.4)	0.030^*b*^
ALT (0–40 U/L)	47.0 (21.0, 113.0)	45.0 (21.0, 109.1)	52.0 (21.0, 156.0)	0.317	40.5 (20.3, 97.0)	63.0 (21.0, 134.0)	0.063
TBIL (3.4–17.7 µmol/L)	14.8 (9.7, 32.2)	14.1 (9.7, 26.4)	24.0 (9.1, 61.6)	0.021^*b*^	13.8 (9.0, 32.5)	17.1 (11.1, 31.5)	0.113
ALB (39–54 g/L)	30.6 ± 6.7	31.4 ± 6.4	27.5 ± 6.9	<0.001^*b*^	29.8 ± 6.7	32.1 ± 6.4	0.003^*b*^
Scr (27–66 µmol/L)	29.4 (20.2, 50.6)	27.3 (19.8, 44.8)	47.0 (28.0, 81.4)	<0.001^*b*^	25.6 (18.2, 47.5)	34.0 (25.0, 53.6)	<0.001^*b*^
BUN (2.7–7 mmol/L)	5.9 (4.0, 8.9)	5.3 (3.8, 8.4)	8.0 (5.5, 12.1)	<0.001^*b*^	5.5 (3.8, 8.5)	6.4 (4.2, 9.4)	0.080
PH (7.35–7.45)	7.39 (7.30, 7.45)	7.40 (7.34, 7.46)	7.28 (7.11, 7.37)	<0.001^*b*^	7.39 (7.30, 7.45)	7.37 (7.29, 7.44)	0.287
BE (−3–+3)	16.4 ± 7.7	5.1 ± 6.7	11.3 ± 9.0	<0.001^*b*^	6.3 ± 7.4	6.8 ± 8.5	0.609
LAC (0.5–2.2 mmol/L)	2.2 (1.4, 3.6)	1.9 (1.3, 2.9)	4.2 (2.2, 9.5)	<0.001^*b*^	2.1 (1.4, 3.3)	2.3 (1.4, 4.3)	0.272
APTT (20–44 s)	40.3 (34.3, 51.4)	39.1 (33.7, 47.8)	52.5 (39.9, 71.1)	<0.001^*b*^	40.5 (34.8, 51.7)	40 (33.5, 51.3)	0.471
Fib (2–4 g/L)	3.6 (2.1, 5.1)	3.6 (2.2, 5.2)	2.7 (1.5, 4.4)	0.019^*b*^	4.0 (2.4, 5.6)	2.7 (1.9, 4.2)	<0.001^*b*^
INR (0.8–1.3)	1.2 (1.1, 1.5)	1.2 (1.0, 1.4)	1.5 (1.3, 1.9)	<0.001^*b*^	1.2 (1.1, 1.5)	1.2 (1.1, 1.4)	0.705
Complications, *n* (%)							
Coagulation function	155 (49.8)	106 (42.7)	49 (77.8)	<0.001^*b*^	101 (50.5)	54 (48.6)	0.754
Septic shock	161 (51.8)	103 (41.5)	58 (92.1)	<0.001^*b*^	95 (47.5)	66 (59.5)	0.043^*b*^
Respiratory failure	224 (72.0)	170 (68.5)	55 (87.3)	0.002^*b*^	163 (81.5)	61 (55.0)	<0.001^*b*^
Brain dysfunction	78 (25.1)	42 (16.9)	36 (57.1)	<0.001^*b*^	47 (23.5)	31 (27.9)	0.388
Acute liver failure	41 (13.2)	21 (8.5)	20 (31.7)	<0.001^*b*^	27 (13.5)	14 (12.6)	0.825
AKI	81 (26.0)	46 (18.5)	37 (58.7)	<0.001^*b*^	49 (24.5)	34 (30.6)	0.140
Treatment, *n* (%)							
MV	146 (46.9)	90 (36.3)	56 (88.9)	<0.001^*b*^	96 (48.0)	45 (40.5)	0.330
CRRT	5 (1.6)	4 (1.6)	1 (1.6)	1.000	3 (1.5)	2 (1.8)	0.839
ECMO	1 (0.3)	1 (0.4)	0 (0.0)	1.000	0 (0)	1 (0.9)	0.357

^*a*^ LOS, length of stay; P/F value, PaO_2_/FiO_2_; pSOFA, pediatric sequential organ failure assessment; WBC, white blood cell; NC, neutrophil count; LC, lymphocyte count; PLT, platelet; Hb, hemoglobin; CRP, C-reactive protein; PCT, procalcitonin; ALT, alanine transaminase; TBIL, total bilirubin; ALB, albumin; Scr, serum creatinine; BUN, blood urea nitrogen; BE, base excess; LAC, lactate; APTT, activated partial thromboplastin time; Fib, fibrinogen; INR, international normalized ratio; AKI, acute kidney injury; MV, mechanical ventilation; CRRT, continuous renal replacement therapy; ECMO, extracorporeal membrane oxygenation; underlying conditions, including congenital heart disease, primary and secondary immunodeficiency, inherited metabolic diseases, leukemia, solid tumors, surgical procedures, and other conditions; /, no data.

^*b*^ *P *< 0.05.

The P:F ratio, PCIS, white blood cell (WBC) count, neutrophil count (NC), platelet count, albumin level, and pH in the non-survival group were lower than in the survival group, while the pSOFA score, PSS, serum creatinine (Scr) level, activated partial thromboplastin time, base excess, lactate level, proportion who had septic shock, brain dysfunction, and AKI were significantly higher (*P* < 0.05). Patients in the HAS group were older, had a higher incidence of underlying diseases and septic shock, and a lower incidence of respiratory failure compared to the CAS group (*P* < 0.05). CAS group patients had a lower WBC count, NC, platelet count, and CRP, PCT, and fibrinogen levels than the HAS group patients, while the albumin and Scr levels were higher.

### Predictive risk factors for 28-day in-hospital mortality

The risk factors for 28-day mortality were PSS (OR = 2.326, 95% CI: 1.811–2.988, *P* < 0.001), NC (OR = 0.896, 95% CI: 0.842–0.954, *P* < 0.001), AKI (OR = 4.630, 95% CI: 1.819–11.782, *P* = 0.001), and MV (OR = 6.100, 95% CI: 2.081–17.881, *P* < 0.001) based on multivariate analysis (Table 2). The risk factors for CAS were consistent with those of the entire cohort. The PSS (OR = 2.238, 95% CI: 1.545–3.240, *P* < 0.001) and AKI (OR = 7.170, 95% CI: 1.624–31.653, *P* = 0.009) were risk factors for HAS. The internal validation of the model through bootstrapping showed good model stability (Table S1). The Hosmer-Lemeshow goodness-of-fit test showed non-significant values for the entire cohort (*χ*² = 3.000, d*f* = 8, *P* = 0.934), the HAS group (*χ*² = 4.026, d*f* = 7, *P* = 0.777), and the CAS group (*χ*² = 6.446, d*f* = 8, *P* = 0.597), which indicated adequate model calibration.

**TABLE 2:** Binary logistic regression analysis of risk factors for 28-day mortality in 311 children with BSI^*a*^

Variables	Total				HAS				CAS			
	Univariate analysis		Multivariate analysis		Univariate analysis		Multivariate analysis		Univariate analysis		Multivariate analysis	
	OR (95% CI)	*P* value	OR (95% CI)	*P* value	OR (95% CI)	*P* value	OR (95% CI)	*P* value	OR (95% CI)	*P* value	OR (95% CI)	*P* value
PSS	2.560 (2.045–3.203)	<0.001^*b*^	2.326 (1.811–2.988)	<0.001^*b*^	2.253 (1.620–3.134)	<0.001^*b*^	2.238 (1.545–3.240)	<0.001^*b*^	2.921 (2.114–4.037)	<0.001^*b*^	2.697 (1.875–3.880)	<0.001^*b*^
NC	0.948 (0.908–0.989)	0.013^*b*^	0.896 (0.842–0.954)	<0.001^*b*^	/	/			0.948 (0.902–0.996)	0.035^*b*^	0.919 (0.856–0.986)	0.019^*b*^
Platelet	0.996 (0.994–0.998)	<0.001^*b*^			/	/			0.996 (0.994–0.999)	0.001^*b*^		
Albumin	0.906 (0.864–0.950)	<0.001^*b*^			/	/			0.913 (0.862–0.967)	0.002^*b*^		
Lactate	1.392 (1.247–1.553)	<0.001^*b*^			/	/			1.499 (1.283–1.752)	<0.001^*b*^		
Septic shock	16.33 (6.329–42.132)	<0.001^b^^*^			18.587 (2.387–144.735)	0.005^*b*^			13.333 (4.967–35.794)	<0.001^*b*^		
AKI	6.249 (3.447–11.331)	<0.001^*b*^	4.630 (1.819–11.782)	0.001^*b*^	11.368 (3.667–35.240)	<0.001^*b*^	7.170 (1.624–31.653)	0.009^*b*^	5.044 (2.437–10.442)	<0.001^*b*^	3.806 (1.134–12.771)	0.030^*b*^
MV	7.460 (3.780–14.728)	<0.001^*b*^	6.100 (2.081–17.881)	<0.001^*b*^	6.100 (2.026–18.371)	0.001^*b*^			8.314 (3.479–19.871)	<0.001^*b*^	5.890 (1.447–23.977)	0.013^*b*^

^*a*^ PSS, Phoenix Sepsis Score; NC, neutrophil count; AKI, acute kidney injury; MV, mechanical ventilation; /, indicates that the corresponding variable was not included in the specified analysis.

^*b*^ Indicates statistically significant results, *P* < 0.05.

**Fig 1:**
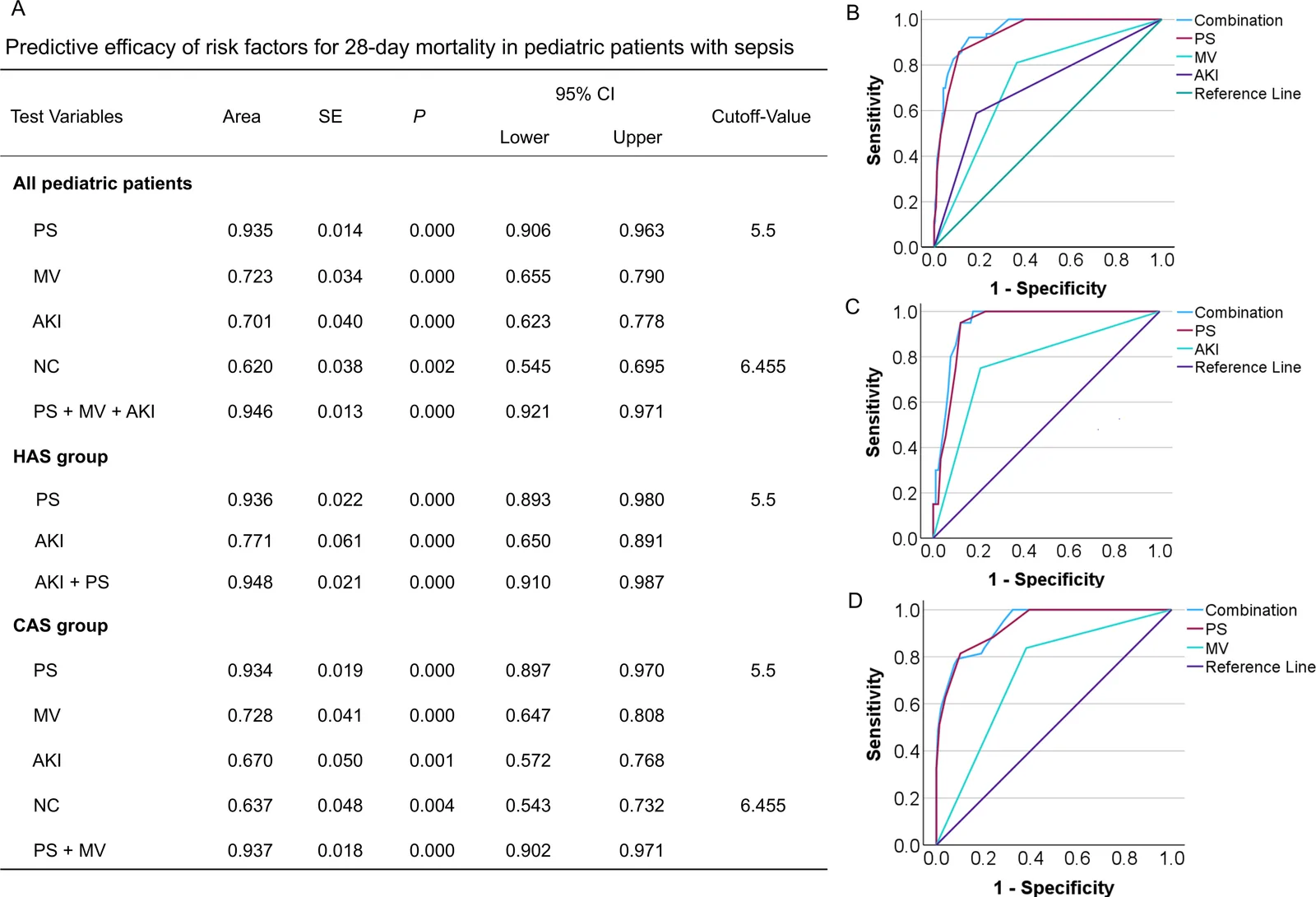
Predictive efficacy of risk factors for 28-day mortality in pediatric patients with sepsis. (**A**) The predictive performance of risk factors for 28-day mortality across three cohorts. (**B**) ROC curve analysis of risk factors in the cohort. (**C**) ROC analysis of risk factors in the HAS group; (**D**) ROC analysis of risk factors in the CAS group. PSS, Phoenix Sepsis Score; MV, mechanical ventilation; AKI, acute kidney injury; NC, neutrophil count.

ROC curve analysis revealed that the model combining PSS with MV and AKI exhibited the best predictive performance (AUC = 0.946, 95% CI: 0.921–0.971) with a sensitivity of 92.1% and a specificity of 84.7%. The predictive efficacy of PSS combined with AKI was excellent in the HAS group (AUC = 0.948, 95% CI: 0.910–0.987) with a sensitivity of 95.0% and a specificity of 87.9%. The AUC of the combined PSS and MV model was 0.937 in the CAS group (95% CI: 0.902–0.971) with a sensitivity of 79.1% and a specificity of 91.1% (Fig. 1). The Hosmer-Lemeshow goodness-of-fit test indicated no evidence of poor calibration. The results of the bootstrap internal validation are presented in Table S1.

### Microbiologic investigations

**Fig 2:**
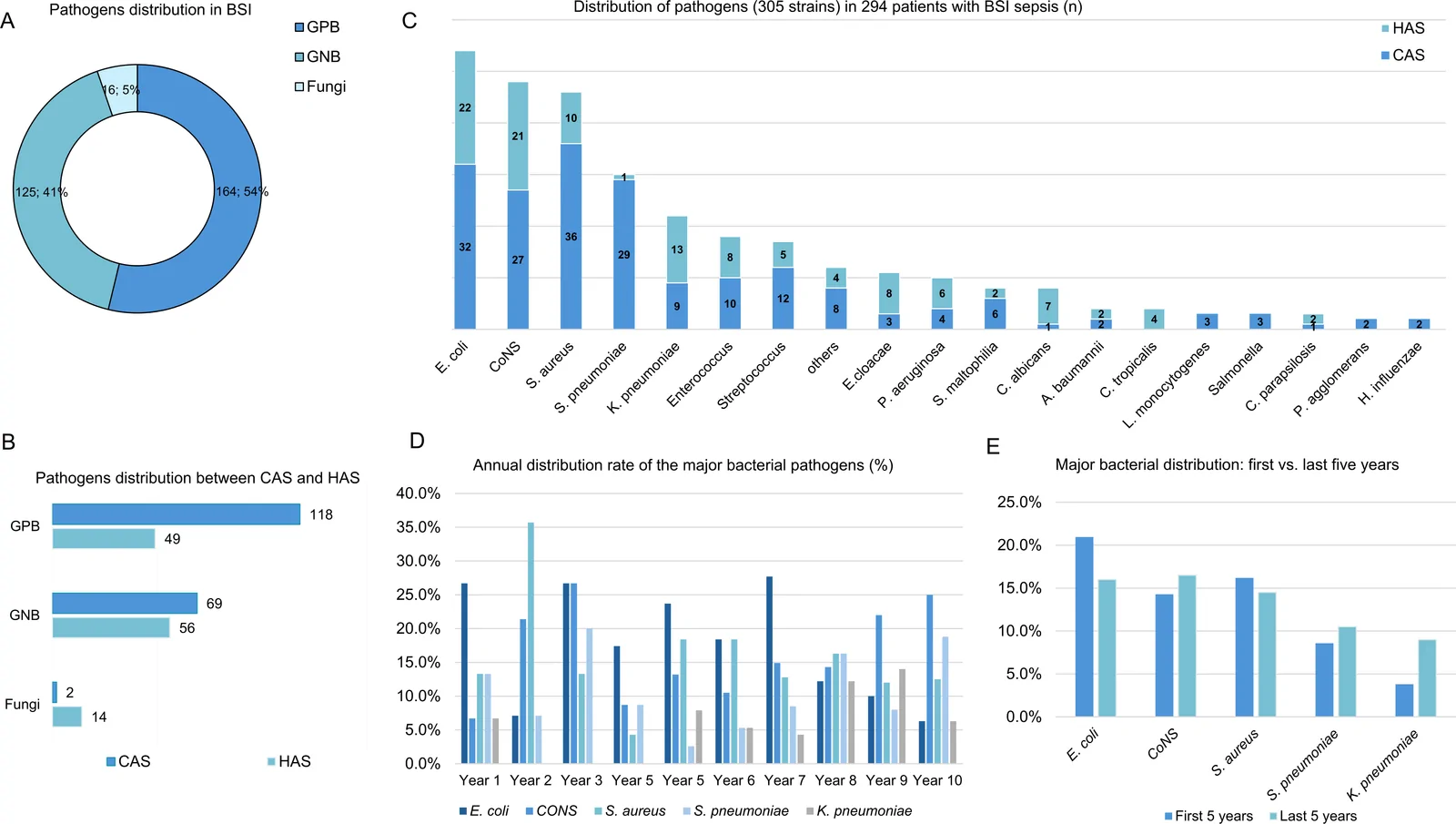
Frequency and distribution of bacteria (305 strains) isolated from 294 sepsis patients investigated for BSI in the PICU. (**A**) Pathogen distribution in BSI. (**B**) Pathogen distribution between the CAS and HAS groups. (**C**) All organisms isolated in pediatric sepsis patients. (**D**) Annual distribution rate of the major bacterial pathogens. (**E**) Major bacterial distribution: first vs last 5 years. GNB, gram-negative bacteria; GPB, gram-positive bacteria; *E. coli*, *Escherichia coli*; CoNS, coagulase-negative Staphylococcus; *S. aureus*, *Staphylococcus aureus; S. pneumoniae*, *Streptococcus pneumoniae; K. pneumoniae*, *Klebsiella pneumoniae; E. cloacae, Enterobacter cloacae; P. aeruginosa*, *Pseudomonas aeruginosa; S. maltophilia, Stenotrophomonas maltophilia; C. albicans*, *Candida albicans; A. baumannii*, *Acinetobacter baumannii; C. tropicalis, Candida tropicalis; L. monocytogenes*, *Listeria monocytogenes; C. parapsilosis*, *Candida parapsilosis; P. agglomerans*, *Pantoea agglomerans; H. influenzae*, *Haemophilus influenzae*. Other pathogens included (*n* = 12): one strain each of *Gemella morbillorum*, *Rothia mucilaginosa*, *Citrobacter braakii*, *Achromobacter denitrificans*, *Achromobacter xylosoxidans*, *Haemophilus parainfluenzae*, *Acinetobacter junii*, *Lamonella ovina*, *Burkholderia cepacia*, *Neisseria meningitidis*, *Serratia marcescens*, and *Candida lusitaniae*.

Among the 311 sepsis patients in this study, 17 were excluded due to unknown bacterial species from blood cultures. Ultimately, among the 294 children in the final analysis, 11 had two separate strains isolated simultaneously, and a total of 305 pathogens were eventually isolated. The composition of the 305 pathogen types was mainly gram-positive bacteria (GPB), accounting for 54% (164/305) of isolates (Fig. 2A). GPB was the main type of pathogen detected in the CAS group, while gram-negative bacteria (GNB) were the main type of pathogen detected in the HAS group (Fig. 2B). The most frequently isolated pathogens were *E. coli*, CoNS, and *S. aureus*, accounting for 48.5% of isolates. The main community-dominant bacteria were *S. aureus*, *E. coli*, and *Streptococcus pneumoniae*. Hospital-dominant bacteria were *E. coli*, CoNS, and *K. pneumoniae* (Fig. 2C). The number of *E. coli* exhibited a downward trend over time, while CoNS and *S. pneumoniae* exhibited an upward trend. *S. aureus* had the highest proportion in the second year of the study (from July 2016 to July 2017), and *K. pneumoniae* accounted for a relatively high proportion from July 2022 to July 2024 (Fig. 2D). The *E. coli* detection rate increased compared to the previous 5 years, while *K. pneumoniae* decreased in the following 5 years (*P* < 0.05, Fig. 2E).

### Antimicrobial resistance patterns of bacterial isolates

Among the GPB, CoNS had a high resistance rate to penicillin, oxacillin, erythromycin, and clindamycin, and a moderate resistance rate to gentamicin, levofloxacin, moxifloxacin, trimethoprim-sulfamethoxazole, and rifampicin, as depicted in Table 3. *S*. *aureus* had a high resistance rate to penicillin, erythromycin, and clindamycin. The MRSA detection rate was 38.5%. *S. pneumoniae* exhibited the highest resistance rates to erythromycin (100%) and tetracycline (91.7%). *Enterococcus* had 86.7% resistance to penicillin and 100% resistance to clindamycin. Vancomycin, tigecycline, or linezolid-resistant strains were not isolated among GPB.

**TABLE 3:** Antimicrobial resistance rates of main bacteria isolated from patients investigated for BSI^*a*^

GPB	PEN (%)	OXA (%)	VAN (%)	LZD (%)	ERY (%)	CLI (%)	GEN (%)	LVX (%)	MXF (%)	SXT (%)	RIF (%)	TCY (%)	TGC (%)
CoNS (*n* = 32)	96.9	96.9	0.0	0.0	87.1	60.0	25.8	41.9	32.3	35.5	19.4	NA	0.0
*S. aureus* (*n* = 40)	97.4	38.5	0.0	0.0	71.8	58.9	17.9	7.5	2.5	15.0	5.1	NA	0.0
*S. pneumoniae* (*n* = 26)	16.0	NA	0.0	0.0	100.0	NA	NA	4.0	4.2	60.0	NA	91.7	NA
*Enterococcus* (*n* = 15)	86.7	NA	0.0	0.0	66.7	100.0	46.7	50.0	50.0	37.5	NA	NA	0.0

GNB	AMC (%)	TZP (%)	CRO (%)	CAZ (%)	CFS (%)	FEP (%)	ETP (%)	LVX (%)	IPM (%)	AMK (%)	ATM (%)	SXT (%)	TGC (%)
*E. coli*(*n* = 51)	17.0	6.1	74.0	28.6	13.3	38.8	2.2	46.9	6.0	0.0	65.9	75.0	0.0
*K. pneumoniae*(*n* = 22)	36.4	35.0	50.0	36.4	27.8	31.8	25.0	18.2	27.3	4.5	21.4	52.4	0.0
*E. cloacae*(*n* = 11)	100.0	28.6	63.7	45.5	NA	0.0	0.0	0.0	9.1	0.0	37.5	9.1	NA
*P. aeruginosa*(*n* = 9)	100.0	0.0	80.0	0.0	0.0	0.0	NA	0.0	0.0	0.0	NA	88.9	100.0

^*a*^ PEN, penicillin G; OXA, oxacillin; VAN, vancomycin; LZD, linezolid; ERY, erythromycin; CLI, clindamycin; GEN, gentamicin; LVX, levofloxacin; MXF, moxifloxacin; SXT, trimethoprim-sulfamethoxazole; RIF, rifampicin; TCY, tetracycline; TGC, tigecycline; AMC, amoxicillin-clavulanate; TZP, piperacillin-tazobactam; CRO, ceftriaxone; CAZ, ceftazidime; CFS, cefoperazone-sulbactam; FEP, cefepime; ETP, ertapenem; IPM, imipenem; AMK, amikacin; ATM, aztreonam; SXT, trimethoprim-sulfamethoxazole; NA, not applicable.

Among the GNB, the resistance rates of *E. coli* to ceftriaxone, trimethoprim-sulfamethoxazole, and aztreonam were all >65%, while the resistance rates to ceftazidime (28.6%) and cefoperazone-sulbactam (13.3%) were lower. *E. coli* was sensitive to piperacillin-tazobactam, ertapenem, imipenem, amikacin, and tigecycline (resistance rate <10%). *K. pneumoniae* was highly resistant to ceftriaxone (50%) and tetracycline (52.4%) but sensitive to amikacin (4.5%) and tigecycline (0%). The resistance rates of *Enterobacter cloacae* to amoxicillin-clavulanate, ceftriaxone, and ceftazidime were 100.0%, 63.7%, and 45.5%, respectively. *E. cloacae* was sensitive to cefepime, ertapenem, levofloxacin, imipenem, amikacin, and trimethoprim-sulfamethoxazole (resistance rate <10%). *P. aeruginosa* had a high resistance rate (≥80%) to amoxicillin-clavulanate, ceftriaxone, trimethoprim-sulfamethoxazole, and tigecycline but was sensitive to piperacillin-tazobactam, ceftazidime, cefoperazone-sulbactam, cefepime, levofloxacin, imipenem, and amikacin with resistance rates all 0.0%.

### Discrepancy in bacterial resistance patterns between the CAS and HAS groups

**Fig 3:**
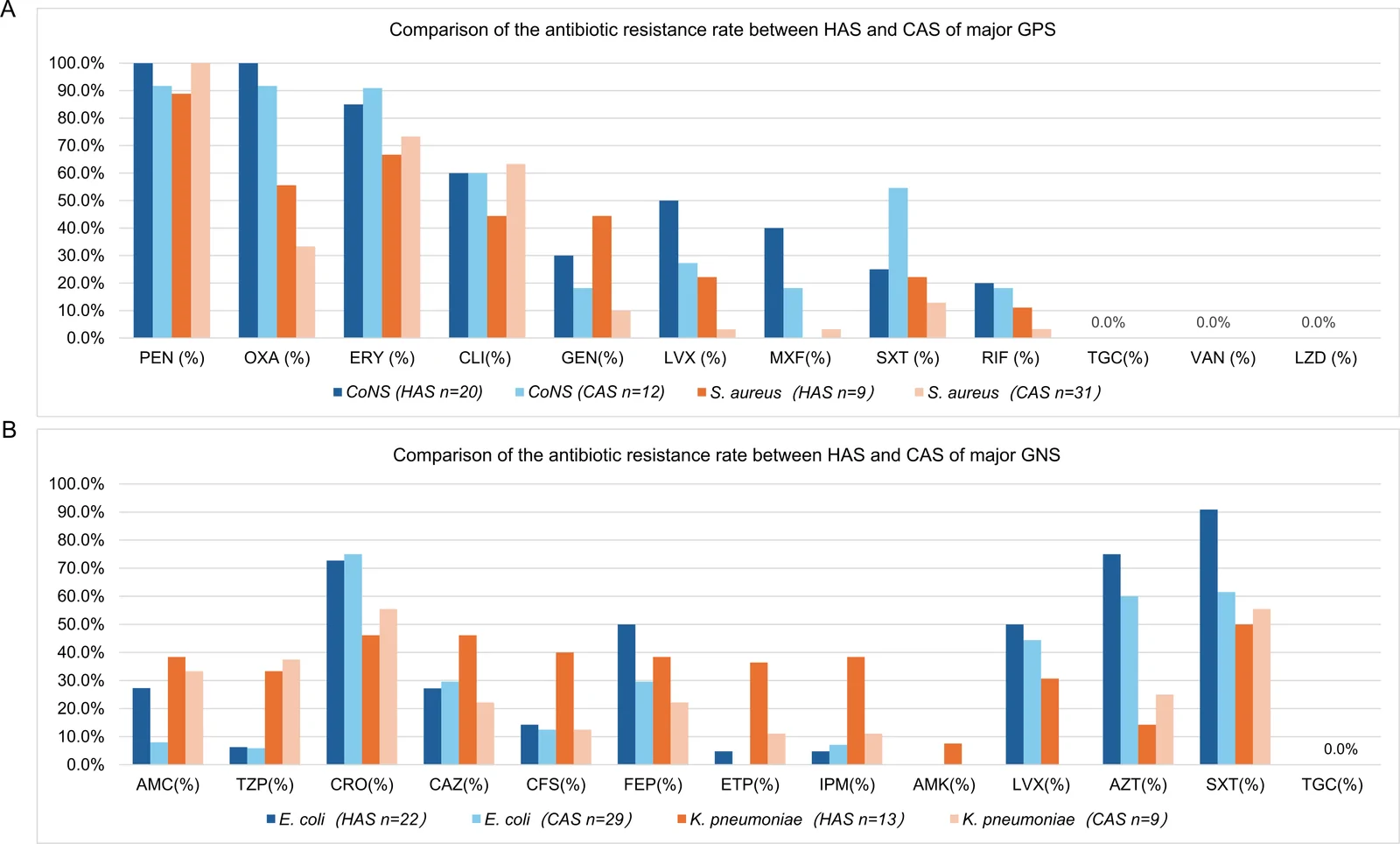
Comparison of the antibiotic resistance rate of major GPB and GNB between the HAS and CAS groups. (**A**) *E. coli* and *K. pneumoniae*. (**B**) CoNS and *S. aureus*.

There were differences in antibiotic resistance between the CAS and HAS groups. The CoNS resistance rates to penicillin, oxacillin, and erythromycin were ≥85% in both groups. The CoNS resistance rates to penicillin, oxacillin, gentamicin, levofloxacin, and moxifloxacin were higher in the HAS group, while the CoNS resistance rate to trimethoprim-sulfamethoxazole was higher in the CAS group. The *S. aureus* resistance rates to penicillin, erythromycin, and clindamycin were higher in the CAS group than the HAS group, while the *S. aureus* resistance rates to oxacillin, gentamicin, levofloxacin, trimethoprim-sulfamethoxazole, and rifampicin were lower in the CAS group than the HAS group. *S. aureus* and CoNS were sensitive to vancomycin, linezolid, and tigecycline in both groups, and no resistant strains were detected (Fig. 3A). The *E. coli* resistance rates to amoxicillin-clavulanate, cefepime, aztreonam, and trimethoprim-sulfamethoxazole were all higher in the HAS group compared to the CAS group, and the *K. pneumoniae* resistance rates to ceftazidime, cefoperazone-sulbactam, cefepime, ertapenem, imipenem, and levofloxacin were also higher. Both *E. coli* and *K. pneumoniae* were sensitive to piperacillin-tazobactam, ertapenem, imipenem, amikacin, and tigecycline in both groups, with resistance rates <10% (Fig. 3B).

### Distribution of antimicrobial resistance phenotypes in major GNB

**Fig 4:**
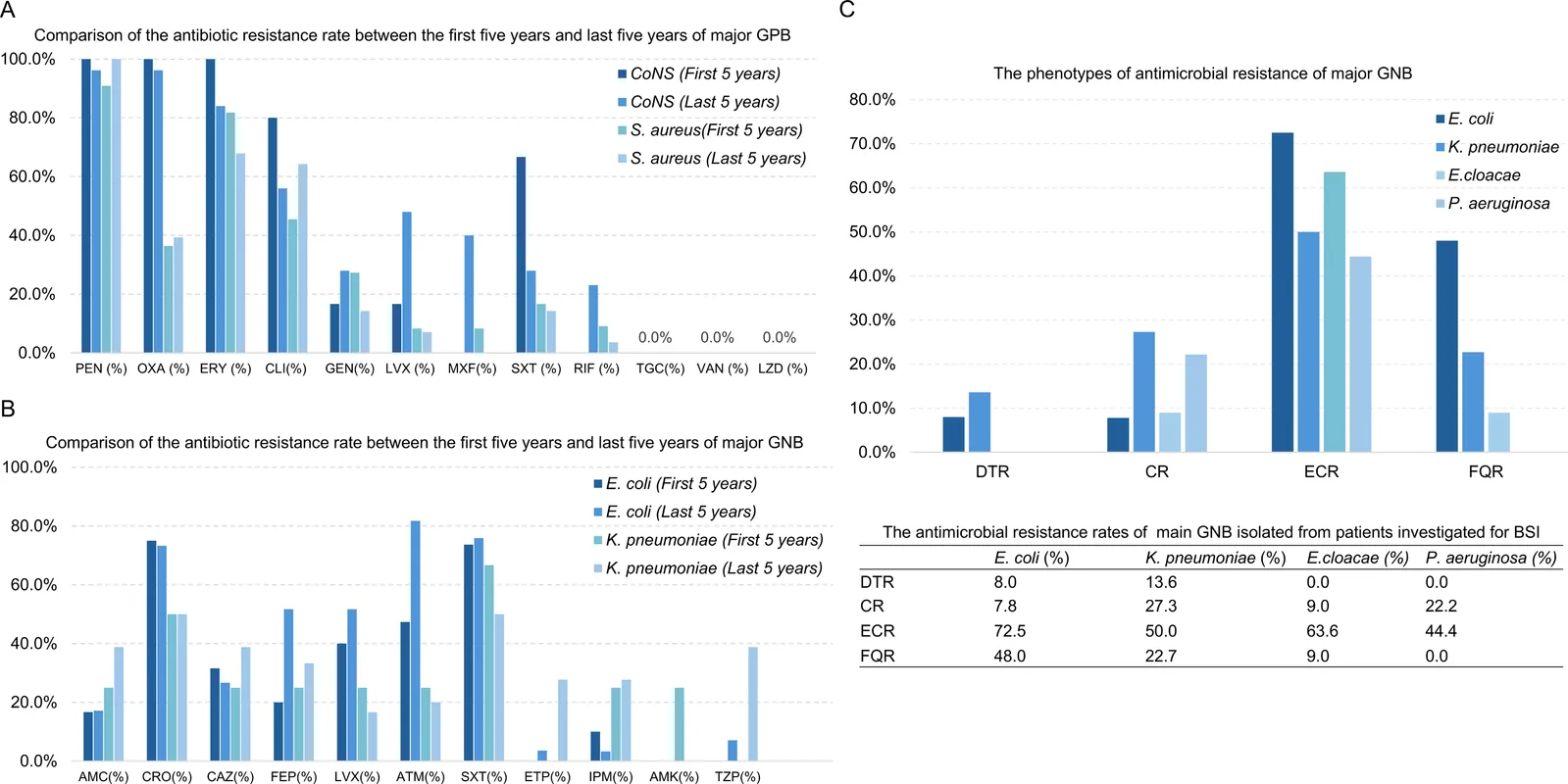
Antimicrobial resistance in major bacteria: rates and GNB phenotypes across the decade. (**A**) GPB resistance rates: first vs last 5 years. (**B**) GNB resistance rates: first vs last 5 years. (**C**) Antimicrobial resistance phenotypes of major GNB. PEN, penicillin G; OXA, oxacillin; VAN, vancomycin; LZD, linezolid; ERY, erythromycin; CLI, clindamycin; GEN, gentamicin; LVX, levofloxacin; MXF, moxifloxacin; SXT, trimethoprim-sulfamethoxazole; RIF, rifampicin; TCY, tetracycline; TGC, tigecycline; AMC, amoxicillin-clavulanate; TZP, piperacillin-tazobactam; CRO, ceftriaxone; CAZ, ceftazidime; CFS, cefoperazone-sulbactam; FEP, cefepime; ETP, ertapenem; IPM, imipenem; AMK, amikacin; ATM, aztreonam; SXT, trimethoprim-sulfamethoxazole; NA, not applicable. CR, carbapenem resistance; DTR, difficult-to-treat resistance; ECR, extended-spectrum cephalosporin resistance; FQR, fluoroquinolone resistance.

Analysis of the drug-resistant phenotypes of *E. coli*, *K. pneumoniae*, *E. cloacae*, and *P. aeruginosa* indicated that ECR was the most predominant drug-resistant phenotype, as shown in Figure 4. The ECR (72.5%) and FQR detection rates (48%) in *E. coli* were the highest, while the CR rate was the lowest (7.8%). The ECR (44.4%) and FQR detection rates (0%) in *P. aeruginosa* were the lowest among the four strains, while the CR rate in *K. pneumoniae* was the highest among the four strains (27.3%). The DTR drug-resistant phenotype was rare. In fact, only *K. pneumoniae* (13.6%) and *E. coli* (8.0%) were detected, and >80% were isolated from the HAS group patients.

## DISCUSSION

A 10-year retrospective analysis was performed from a single hospital to investigate BSI-induced sepsis in children. The 28-day in-hospital mortality rate was 20.3% lower than that previously reported for BSI-induced severe sepsis in PICUs from Tianjin and Shanghai, China (36.98% and 28.8%, respectively) (14, 15). There were some discrepancies in clinical features between the CAS and HAS groups. Patients in the HAS group were older, had a higher mortality rate, and a higher incidence of underlying diseases and septic shock, but a lower incidence of respiratory failure compared to the CAS group. Patients in the CAS group had a lower WBC count, NC, platelet count, and CRP, PCT, and fibrinogen levels, but higher albumin and Scr levels than the HAS group.

The risk factors for 28-day mortality were the PSS, a decreased NC, AKI, and MV. The risk factors for mortality in the CAS group were consistent with the entire cohort. PSS and AKI were risk factors for mortality in the HAS group. A previous study reported poor prognosis in children with severe sepsis associated with the pSOFA score and P:F ratio (14), whereas in the current study, poor prognosis was associated with the PSS based on the new international consensus criteria for pediatric sepsis. A previous study showed that neutropenia was one of the factors that could increase the risk of infection (16). A reduced NC was also identified in the current study as an important risk factor. Existing evidence has confirmed that patients with AKI are at high risk of poor outcomes (17, 18), which was also demonstrated in the current study. Like previous studies (19, 20), MV was related to a poor prognosis.

A total of 305 pathogenic bacteria (predominantly GPB [54%]) were isolated from blood cultures obtained from pediatric patients with sepsis in the current study, while a previous study reported GNB dominance (14). GPB was the main pathogen type detected in the CAS group, while GNB was the main pathogen type detected in the HAS group. The most frequently isolated pathogens were *E. coli*, CoNS, and *S. aureus*, which is consistent with results from previous studies (5, 15). The main community-dominant bacteria were *S. aureus*, *E. coli*, and *S. pneumoniae*. The hospital-dominant bacteria were *E. coli*, CoNS, and *K. pneumoniae*.

Multiple surveys have shown that the number of cases involving MDR BSIs has been on the rise over the past period of time, especially GNB (5, 21, 22). The drug resistance patterns of the main bacterial isolates were analyzed in the current study. Indeed, all the main GPBs were shown to be susceptible to vancomycin, tigecycline, or linezolid, as reported in a previous study (23). CoNS had a high resistance rate to penicillin, oxacillin, erythromycin, and clindamycin. *S. aureus* exhibited frequent resistance to penicillin and erythromycin, and the MRSA detection rate was 38.5%. In clinical practice, glycopeptides are the preferred treatment for MRSA, whereas beta-lactam antibiotics are preferred for methicillin-susceptible *S. aureus* because of the poor therapeutic effect of glycopeptides (24, 25). Infections caused by resistant GNB are becoming increasingly concerning (22). GNB was predominant in the current study. *E. coli* had high resistance to ceftriaxone, trimethoprim-sulfamethoxazole, and aztreonam but was sensitive to piperacillin-tazobactam, ertapenem, imipenem, amikacin, and tigecycline, which is consistent with the results from other studies (15, 26). *K. pneumoniae* was shown to be highly resistant to ceftriaxone and tetracycline. An alarmingly high resistance rate was noted to ertapenem and imipenem (>25%), which is higher than the meropenem resistance rate reported in previous studies (14, 26). This finding suggests that *K. pneumoniae* resistance to carbapenem antibiotics is gradually developing, but corollary studies to determine the trajectory of resistance in this geographic region are warranted for an accurate assessment.

ECR was the most common GNB drug-resistant phenotype among *E. coli*, *K. pneumoniae*, *E. cloacae*, and *P. aeruginosa*. The detection rate for CR-*E. coli* was 7.8% in the current study, which was lower than the detection rate for CR-*K. pneumoniae* (27.3%). The detection rates herein were lower compared to the detection rates reported by Lyu et al. (23). The ECR and FQR detection rates in *K. pneumoniae* were lower than those in *E. coli*, which were consistent with the findings in a previous study (14). The ECR and FQR detection rates in *E. coli* were the highest, as were the CR and DTR rates in *K. pneumoniae. K. pneumoniae* was shown to have the highest ECR level, and *E. coli* had the highest FQR level, according to a previous study (23).

Unlike individual patient pathogen susceptibility results, which become available only after 48–72 h and guide targeted therapy, our regional resistance data provide actionable information at the point of clinical suspicion. These findings enable clinicians to achieve the following: (i) perform early risk stratification by identifying patients likely to harbor resistant organisms; (ii) select empirical antibiotic regimens that reflect current local resistance patterns, thereby reducing the risk of inappropriate initial therapy; and (iii) design antimicrobial stewardship interventions tailored to regional epidemiology. Therefore, while individual susceptibility results are essential for definitive treatment, the regional data directly support improved pediatric sepsis outcomes by shortening the time to effective therapy, which is a key determinant of survival. This finding fills the regional knowledge gap and has direct implications for clinical practice, as originally stated in the Introduction.

Additionally, a small number of patients (<2%) were excluded due to insufficient data for retrospective PSS calculation. Although the proportion was very low and missing data appeared to be random (related to documentation rather than clinical severity), we cannot completely exclude the possibility of minor selection bias. Prospective validation with complete data collection is warranted.

### Conclusion

Higher mortality among children with BSI-induced sepsis was shown to be associated with an elevated PSS and reduced NC, as well as AKI and MV. The pathogens and antibiotic resistance in BSI-induced sepsis vary in different geographic regions. Therefore, it is essential to monitor the MDR in the local area and select antibiotics based on the results of drug sensitivity tests.

## Data Availability

All data generated during this study are included in this published article and its supplemental material.

## SUPPLEMENTAL MATERIAL

Figure S1 legend spectrum.00194-26-s0001.docx Figure S1 legend.

Figure S1 spectrum.00194-26-s0002.tif Flowchart of patients’ enrollment and study design in BSI pediatric sepsis.

Table S1 spectrum.00194-26-s0003.docx Multivariate binary logistic regression model with bootstrap internal validation for 28-day mortality in 311 children with bloodstream infection (BSI).

ASM does not own the copyrights to Supplemental Material that may be linked to, or accessed through, an article. The authors have granted ASM a non-exclusive, world-wide license to publish the Supplemental Material files. Please contact the corresponding author directly for reuse.
