# Supplementary material for: Pathogenic spectrum, antibiotic resistance, and risk factors for 28-day mortality in pediatric bloodstream infection-associated sepsis: a 10-year retrospective cohort study
Source: Microbiol Spectr. 2026 Jul 20;14(9):e00194-26. doi: 10.1128/spectrum.00194-26 (PMC13532225; doi:10.1128/spectrum.00194-26)
Supplement: Supplementary file 1 [file spectrum.00194-26-s0001.docx]

Figure S1. Flowchart of patient enrollment and study design in BSI-induced pediatric sepsis. PICU, pediatric intensive care unit; HAS, hospital-acquired sepsis, CAS, community-acquired sepsis
