# Supplementary material for: Pathogenic spectrum, antibiotic resistance, and risk factors for 28-day mortality in pediatric bloodstream infection-associated sepsis: a 10-year retrospective cohort study
Source: Microbiol Spectr. 2026 Jul 20;14(9):e00194-26. doi: 10.1128/spectrum.00194-26 (PMC13532225; doi:10.1128/spectrum.00194-26)
Supplement: Supplementary file 2 [file spectrum.00194-26-s0003.docx]

Supplementary Table 1. Multivariate binary logistic regression model with bootstrap internal validation for 28-day mortality in 311 children with bloodstream infection (BSI)

| Variables | Total cohort | | | |  | HAS | | | | | |  | CAS | | | |
| --- | --- | --- | --- | --- | --- | --- | --- | --- | --- | --- | --- | --- | --- | --- | --- | --- |
|  | OR (95％CI) | OR (95％CI) (Corrected) | *P* | *P-*Corrected |  | OR (95％CI) | OR (95％CI) (Corrected) | | *P* | *P-*Corrected | |  | OR (95％CI) | OR (95％CI) (Corrected) | *P* | *P-*Corrected |
| PSS | 2.326(1.811-2.988) | 2.326(1.878-3.387) | ＜0.001 | ＜0.001 |  | 2.238（1.545-3.240） | | 2.238(1.709-4.237) | ＜0.001 | | ＜0.001 |  | 2.697(1.875-3.880) | 2.697(2.121-4.948) | ＜0.001 | ＜0.001 |
| NC | 0.896(0.842-0.954) | 0.896(0.796-0.946) | ＜0.001 | ＜0.001 |  |  | |  |  | |  |  | 0.919(0.856-0.986) | 0.919(0.794-0.971) | 0.019 | 0.017 |
| AKI | 4.630(1.819-11.782) | 4.630(1.885-15.02) | 0.001 | 0.003 |  | 7.170(1.624-31.653) | | 7.170(1.523-98.28) | 0.009 | | 0.002 |  | 3.806(1.134-12.771) | 3.806(1.212-23.71) | 0.030 | 0.033 |
| MV | 6.100(2.081-17.881) | 6.100(2.452-23.76) | ＜0.001 | ＜0.001 |  |  | |  |  | |  |  | 5.890(1.447-23.977) | 5.890(1.689-45.93) | 0.013 | 0.004 |

Note. *P* < 0.05 indicates statistical significance. Bootstrap-corrected OR/P-values from internal validation; Blanks denote variables excluded from the final model; PSS, Phoenix sepsis score.
